# Supplementary material for: Analysis of PPARγ Signaling Activity in Psoriasis
Source: Int J Mol Sci. 2021 Aug 10;22(16):8603. doi: 10.3390/ijms22168603 (PMC8395241; doi:10.3390/ijms22168603)
Supplement: Supplementary file 1 [file ijms-22-08603-s001.zip › Supplemental materials_Analysis of PPARg signaling activity in psoriasis/Pathway models/Models images and html files/Differentiation of psoriatic T cells/6190.html]

IFNAR2


# Protein IFNAR2

|  |  |
| --- | --- |
| URN | urn:agi-llid:3455 |
| Total Entities | 0 |
| Connectivity | 336 |
| Name | IFNAR2 |
| Description | interferon alpha and beta receptor subunit 2 |
| Notes | The protein encoded by this gene is a type I membrane protein that forms one of the two chains of a receptor for interferons alpha and beta. Binding and activation of the receptor stimulates Janus protein kinases, which in turn phosphorylate several proteins, including STAT1 and STAT2. Multiple transcript variants encoding at least two different isoforms have been found for this gene. [provided by RefSeq, Jul 2008] |
| Primary Cell Localization | Plasma membrane |
| Class | Receptor |
| ObjectType | Protein |

---

|  |  |
| --- | --- |
| Pathway | AXL Receptor Inhibits Macrophages and Dendritic Cells Function |
|  | SOCS in Negative Feedback Regulation in Myocarditis |
|  | T-Cells Differentiation Block in Psoriasis |
|  | Dendritic Cell Activation in Systemic Lupus Erythematosis |
|  | Th17-Cell Function in Systemic Lupus Erythematosus |
|  | B-Cell Activation in Systemic Lupus Erythematosus |
|  | Dendritic Cell Dysfunction in Psoriatic Arthritis |
|  | N2 Neutrophils in Tumor-Promoting Inflammation and Tumor Progression |
|  | IFNW1/IFNR Expression Target |
|  | IFNA1/IFNR Expression Targets |
|  | IFNB1/IFNR Expression Targets |
|  | Elevated Receptors -> Expression Targets in Lymphoid System and Blood |
|  | AXL Receptor Tyrosine Kinase Signaling |
|  | PPAR Psoriasis |
|  | New Pathway (3) |
|  | IFN signaling in alveolar cells |
|  | Dendritic Cell Activation in COVID19 |
|  | 1\_Differentiation of psoriatic T cells |
|  | drug-target pairs binding |
|  | drug-target pairs direct regulation |
|  | Differentiation of psoriatic T cells |

---

|  |  |
| --- | --- |
| MedScan ID | 3455 |

---

|  |  |
| --- | --- |
| LocusLink ID | 3455 |
|  | 15976 |
|  | 686326 |

---

|  |  |
| --- | --- |
| Alias | IFN-R-2 |
|  | interferon alpha/beta receptor 2 |
|  | INR2 |
|  | OTTHUMP00000067675 |
|  | IMD45 locus |
|  | interferon (alpha and beta) receptor II |
|  | IFNABR |
|  | interferon receptor JFNAR 2 II |
|  | OTTHUMP00000067676 |
|  | LOC194555 |
|  | INR2\_HUMAN |
|  | type I interferon receptor 2 |
|  | LOC686326 |
|  | Interferon alpha/beta receptor II |
|  | interferon (alpha, beta and omega) receptor 2 |
|  | interferon alpha and beta receptor subunit 2 |
|  | interferon (alpha and beta) receptor 2 |
|  | type I interferon receptor soluble isoform |
|  | interferon receptor 2c |
|  | type I interferon alpha/beta receptor 2a |
|  | AI747302 |
|  | OTTMUSP00000046708 |
|  | IFNARB |
|  | human interferon alpha/beta receptor |
|  | IFNAR2 |
|  | interferon-alpha/beta receptor beta chain |
|  | OTTHUMP00000206687 |
|  | OTTHUMP00000067674 |
|  | interferon receptor 2a' |
|  | OTTMUSP00000033777 |
|  | interferon receptor JFNAR 2-2 |
|  | IFN-alpha/beta receptor 2 |
|  | OTTMUSP00000046710 |
|  | interferon receptor 2 |
|  | interferon (alpha, beta and omega) receptor II |
|  | IFN-R |
|  | IMD45 |
|  | IFN-alpha-REC |
|  | interferon alpha binding protein |
|  | interferon receptor |
|  | Ifnar-2 |
|  | IFN-alpha binding protein |
|  | interferon-alpha/beta receptor 2 |

---

|  |  |
| --- | --- |
| GO ID | 0004896 |
|  | 0019901 |
|  | 0004905 |
|  | 0007259 |
|  | 0007166 |
|  | 0019221 |
|  | 0051607 |
|  | 0060338 |
|  | 0035455 |
|  | 0035456 |
|  | 0009615 |
|  | 0060337 |
|  | 0005576 |
|  | 0005887 |
|  | 0005886 |
|  | 0019962 |
|  | 0008283 |
|  | 0006357 |
|  | 0005615 |
|  | 0016021 |
|  | 0016020 |
|  | 0005622 |
|  | 0005515 |
|  | 0042015 |

---

|  |  |
| --- | --- |
| KEGG ID | hsa:3455 |
|  | mmu:15976 |

---

|  |  |
| --- | --- |
| Organism | Homo sapiens {Organism urn:agi-taxid:9606} |
|  | Mus musculus {Organism urn:agi-taxid:10090} |
|  | Rattus norvegicus {Organism urn:agi-taxid:10116} |
|  | Mus musculus |
|  | Homo sapiens |
|  | Rattus norvegicus |

---

|  |  |
| --- | --- |
| Mouse chromosome position | 16 52.82 cM |
|  | 16 63.1 cM |

---

|  |  |
| --- | --- |
| OMIM ID | 602376 |
|  | 610424 |
|  | 616669 |

---

|  |  |
| --- | --- |
| Rat chromosome position | 11q11 |
|  | 11 |

---

|  |  |
| --- | --- |
| Hugo ID | 5433 |
|  | HGNC:5433 |

---

|  |  |
| --- | --- |
| Human chromosome position | 21q22.11 |

---

|  |  |
| --- | --- |
| Swiss-Prot Accession | P48551 |
|  | Q9BUA0 |
|  | P48551.1 |
|  | O35664 |
|  | Q9D1R7 |
|  | Q923Z6 |
|  | O35664.2 |
|  | M0R8H7 |
|  | A8KAJ4 |
|  | D3DSE8 |
|  | D3DSE9 |
|  | Q15467 |
|  | Q6FHD7 |
|  | O35238 |
|  | O35663 |
|  | O35983 |
|  | Q923Z5 |

---

|  |  |
| --- | --- |
| PIR ID | I39073 |
|  | S59501 |

---

|  |  |
| --- | --- |
| GenBank ID | NC\_000021 |
|  | NM\_001289125 |
|  | NP\_001276054 |
|  | NM\_001289128 |
|  | NP\_001276057 |
|  | NM\_207585 |
|  | NP\_997468 |
|  | NM\_001289126 |
|  | NP\_001276055 |
|  | NM\_000874 |
|  | NP\_000865 |
|  | NM\_207584 |
|  | NP\_997467 |
|  | NG\_016003 |
|  | AF039905 |
|  | AH004970 |
|  | AAB46417 |
|  | AAB46418 |
|  | AAB46419 |
|  | AP000292 |
|  | AP000293 |
|  | AP000294 |
|  | AP000295 |
|  | AP001716 |
|  | AY740397 |
|  | AAU21038 |
|  | CH471079 |
|  | EAX09842 |
|  | EAX09843 |
|  | EAX09844 |
|  | EAX09845 |
|  | EAX09846 |
|  | KF457225 |
|  | AK293059 |
|  | BAF85748 |
|  | BC002793 |
|  | AAH02793 |
|  | BC013156 |
|  | BG715143 |
|  | BI520056 |
|  | BM677386 |
|  | CD110317 |
|  | CR541817 |
|  | CAG46616 |
|  | HG380509 |
|  | CDG37809 |
|  | HY104613 |
|  | L41942 |
|  | AAB46413 |
|  | L41943 |
|  | AAB46414 |
|  | L41944 |
|  | AAB46415 |
|  | U29584 |
|  | AAC50202 |
|  | X77722 |
|  | CAA54785 |
|  | X89772 |
|  | CAA61914 |
|  | X89814 |
|  | CAA61940 |
|  | P48551 |
|  | NC\_000082 |
|  | NM\_010509 |
|  | NP\_034639 |
|  | NM\_001110498 |
|  | NP\_001103968 |
|  | XM\_006522923 |
|  | XP\_006522986 |
|  | XM\_011246100 |
|  | XP\_011244402 |
|  | XM\_017316882 |
|  | XP\_017172371 |
|  | NM\_001347258 |
|  | AC152503 |
|  | AF367979 |
|  | AAK73022 |
|  | AAK73023 |
|  | AAK73024 |
|  | AF440786 |
|  | AAL40944 |
|  | AAL40945 |
|  | CH466602 |
|  | EDL03831 |
|  | EDL03832 |
|  | AF013274 |
|  | AAC53351 |
|  | AF013486 |
|  | AAC53352 |
|  | AK003185 |
|  | BAB22628 |
|  | AK010952 |
|  | BAB27287 |
|  | AK139169 |
|  | BAE23909 |
|  | AK150386 |
|  | BAE29516 |
|  | AK150597 |
|  | BAE29690 |
|  | AK151474 |
|  | BAE30430 |
|  | AK151493 |
|  | BAE30446 |
|  | AK151777 |
|  | AK152878 |
|  | BAE31563 |
|  | AK153317 |
|  | BAE31897 |
|  | AK181436 |
|  | AK195915 |
|  | AK199902 |
|  | AK201656 |
|  | AK205320 |
|  | AK209089 |
|  | AK210331 |
|  | AK212415 |
|  | AK218073 |
|  | AV351897 |
|  | BC003743 |
|  | AAH03743 |
|  | BC071225 |
|  | AAH71225 |
|  | BE691178 |
|  | BE993108 |
|  | BX515017 |
|  | Y09813 |
|  | CAA70943 |
|  | Y09864 |
|  | CAA70991 |
|  | Y09865 |
|  | CAA70992 |
|  | O35664 |
|  | NC\_005110 |
|  | XM\_006248107 |
|  | XP\_006248169 |
|  | XM\_017598091 |
|  | XP\_017453580 |
|  | AC\_000079 |
|  | XM\_006221074 |
|  | XP\_006221136 |
|  | XM\_017604280 |
|  | XP\_017459769 |
|  | AABR07033629 |
|  | AABR07033630 |
|  | AAHX01068276 |
|  | AAHX01068277 |
|  | AAHX01068278 |
|  | CH473989 |
|  | EDM10714 |
|  | L42323 |
|  | AMYH02037734 |
|  | AMYH02037733 |
|  | AMYH02037735 |
|  | ABM87259 |
|  | XP\_005261024 |
|  | XP\_005261023 |
|  | XP\_005261022 |
|  | XP\_005261025 |
|  | AAHY01130185 |
|  | AC\_000038 |
|  | XM\_005260965 |
|  | XM\_005260967 |
|  | XM\_005260966 |
|  | XM\_005260968 |
|  | AC\_000153 |
|  | L42240 |
|  | L42241 |
|  | L42242 |
|  | L42243 |
|  | NC\_018932 |
|  | L42237 |
|  | L42238 |
|  | L42239 |
|  | DQ896260 |
|  | ABBA01057336 |
|  | ABBA01057335 |
|  | ABBA01057334 |
|  | ABM83941 |
|  | DQ893015 |

---

|  |  |
| --- | --- |
| Swiss-Prot ID | INAR2\_HUMAN |
|  | INAR2\_MOUSE |

---

|  |  |
| --- | --- |
| Cell Localization | Cell membrane |
|  | Secreted |
|  | Membrane |
|  | Plasma membrane |

---

|  |  |
| --- | --- |
| Ensembl ID | ENSG00000159110 |
|  | ENSG00000249624 |
|  | ENSP00000384309.2 |
|  | ENST00000404220.7 |
|  | ENSP00000343957.4 |
|  | ENST00000342136.8 |
|  | ENSP00000343289.3 |
|  | ENST00000342101.7 |
|  | ENSP00000371699.3 |
|  | ENST00000382264.7 |
|  | ENSP00000496248.1 |
|  | ENST00000646150.1 |
|  | ENSMUSG00000022971 |
|  | ENSMUSP00000113358.1 |
|  | ENSMUST00000117836.7 |
|  | ENSMUSP00000023693.7 |
|  | ENSMUST00000023693.13 |
|  | ENSMUSP00000086443.6 |
|  | ENSMUST00000089042.6 |
|  | ENSRNOG00000045668 |
|  | ENSRNOP00000065773.2 |
|  | ENSRNOT00000072856.2 |
|  | ENSP00000343957 |
|  | ENST00000382264 |
|  | ENST00000382241 |
|  | ENST00000404220 |
|  | ENSRNOT00000072856 |
|  | ENST00000342101 |
|  | ENSMUST00000023693 |
|  | ENSP00000384309 |
|  | ENSMUSP00000023693 |
|  | ENSMUSP00000113358 |
|  | ENSMUST00000089042 |
|  | ENSP00000371676 |
|  | ENSP00000371699 |
|  | ENSRNOP00000065773 |
|  | ENSMUST00000117836 |
|  | ENSMUSP00000086443 |
|  | ENSP00000343289 |
|  | ENST00000342136 |

---

|  |  |
| --- | --- |
| MGI ID | MGI:1098243 |
|  | 1098243 |

---

|  |  |
| --- | --- |
| RGD ID | 1583177 |

---

|  |  |
| --- | --- |
| Unigene ID | Mm.6834 |
|  | Hs.708195 |

---

|  |  |
| --- | --- |
| Homologene ID | 49242 |

---

|  |  |
| --- | --- |
| Shape | Stick-vertex |

---

|  |  |
| --- | --- |
| IPI ID | IPI00220690 |
|  | IPI00395208 |
|  | IPI00395209 |
|  | IPI00220691 |
|  | IPI00010193 |
|  | IPI00312295 |

---
